# On the relationship of first-episode psychosis to the amphetamine-sensitized state: a dopamine D_2/3_ receptor agonist radioligand study

**DOI:** 10.1038/s41398-019-0681-5

**Published:** 2020-01-08

**Authors:** Ana Weidenauer, Martin Bauer, Ulrich Sauerzopf, Lucie Bartova, Lukas Nics, Sarah Pfaff, Cecile Philippe, Neydher Berroterán-Infante, Verena Pichler, Bernhard M. Meyer, Ulrich Rabl, Patrick Sezen, Paul Cumming, Thomas Stimpfl, Harald H. Sitte, Rupert Lanzenberger, Nilufar Mossaheb, Alexander Zimprich, Pablo Rusjan, Georg Dorffner, Markus Mitterhauser, Marcus Hacker, Lukas Pezawas, Siegfried Kasper, Wolfgang Wadsak, Nicole Praschak-Rieder, Matthäus Willeit

**Affiliations:** 1 Department of Psychiatry and Psychotherapy, Division of General Psychiatry, Medical University of Vienna, Währinger Gürtel 18-20, 1090 Vienna, Austria; 2 Department of Clinical Pharmacology, Medical University of Vienna, Vienna, Austria; 3 Department of Biomedical Imaging and Image Guided Therapy, Medical University of Vienna, Vienna, Austria; 4 School of Psychology and Counseling and IHBI, Queensland University of Technology, Brisbane, Australia; 5 Department of Nuclear Medicine, Inselspital, Bern University Hospital, University of Bern, Bern, Switzerland; 6 Department of Laboratory Medicine, Medical University of Vienna, Vienna, Austria; 7 Institute of Pharmacology, Medical University of Vienna, Vienna, Austria; 8 Department of Psychiatry and Psychotherapy, Division of Social Psychiatry, Medical University of Vienna, Vienna, Austria; 9 Department of Neurology, Medical University of Vienna, Vienna, Austria; 10 Research Imaging Centre, Centre for Addiction and Mental Health, University of Toronto, Toronto, Canada; 11 Center for Medical Statistics, Informatics and Intelligent Systems, Medical University of Vienna, Vienna, Austria; 12 Ludwig Boltzmann Institute Applied Diagnostics, Vienna, Austria

**Keywords:** Molecular neuroscience, Schizophrenia

## Abstract

Schizophrenia is characterized by increased behavioral and neurochemical responses to dopamine-releasing drugs. This prompted the hypothesis of psychosis as a state of “endogenous” sensitization of the dopamine system although the exact basis of dopaminergic disturbances and the possible role of prefrontal cortical regulation have remained uncertain. To show that patients with first-episode psychosis release more dopamine upon amphetamine-stimulation than healthy volunteers, and to reveal for the first time that prospective sensitization induced by repeated amphetamine exposure increases dopamine-release in stimulant-naïve healthy volunteers to levels observed in patients, we collected data on amphetamine-induced dopamine release using the dopamine *D*_2/3_ receptor agonist radioligand [^11^C]-(+)-PHNO and positron emission tomography. Healthy volunteers (*n* = 28, 14 female) underwent a baseline and then a post-amphetamine scan before and after a mildly sensitizing regimen of repeated oral amphetamine. Unmedicated patients with first-episode psychosis (*n* = 21; 6 female) underwent a single pair of baseline and then post-amphetamine scans. Furthermore, T1 weighted magnetic resonance imaging of the prefrontal cortex was performed. Patients with first-episode psychosis showed larger release of dopamine compared to healthy volunteers. After sensitization of healthy volunteers their dopamine release was significantly amplified and no longer different from that seen in patients. Healthy volunteers showed a negative correlation between prefrontal cortical volume and dopamine release. There was no such relationship after sensitization or in patients. Our data in patients with untreated first-episode psychosis confirm the “endogenous sensitization” hypothesis and support the notion of impaired prefrontal control of the dopamine system in schizophrenia.

## Introduction

Several lines of evidence demonstrate increased subcortical dopamine (DA) transmission in psychotic patients with schizophrenia (SCZ). Positron emission tomography (PET) studies show increased dopamine synthesis capacity and heightened behavioral and neurochemical responses towards DA-releasing compounds^1–6^. The common mechanism of action of all antipsychotic drugs—reducing DA transmission at postsynaptic D_2/3_ receptors—confirms the key role of DA signaling in psychosis^7^. While the pathophysiological basis of DA dysfunction in SCZ remains unknown, current versions of the DA theory of SCZ^8^ posit that upstream pathogenic factors converge on subcortical DA pathways to mediate the expression and intensity of psychotic symptoms^9–11^.

Sensitization denotes a process by which repeated exposure to a stimulus induces a progressive increase in responses to the very same stimulus^12,13^. When repeatedly administered, d-amphetamine (AMPH) induces behavioral sensitization and a progressive amplification in AMPH-induced DA release^14–16^. Since psychotic patients show elevated responses to AMPH without any prior drug exposure^1^, psychosis has been conceptualized as a state of “endogenous sensitization”^10,17,18^. The prefrontal cortex (PFC), origin of reciprocal regulatory connections to subcortical DA neurons^19^, has often been found to be structurally and functionally impaired in SCZ^20,21^. Earlier studies have shown particularly strong relationships between subcortical DA metabolism and the left-hemispheric dorso-lateral PFC (DLPFC) and inferior frontal gyrus (IFG) in SCZ and subjects at-risk mental state for SCZ^22,23^. In order to collect experimental evidence supporting this concept, we used PET and the DA D_2/3_ receptor agonist radioligand (+)-4-propyl-3,4,4a,5,6,10b-hexahydro-2*H*-naphtho[1,2-b][1,4]oxazin-9-ol ([^11^C]-(+)-PHNO)^24^ for measuring AMPH-induced changes in D_2/3_ receptor binding, semi-quantitative index of DA release, in drug-naïve patients with first-episode psychosis (FEP). Healthy volunteers (HV) were studied before and after exposure to a mildly sensitizing regime of repeated AMPH administration. In order to identify upstream pathogenic mechanisms of psychotic hyperdopaminergia, we analyzed volumetric parameters in the PFC for their relationship to indices of subcortical DA release.

## Materials and methods

All procedures of this study (Clinical Trial Registry: EUDRACT 2010-019586-29) were approved by the ethics committee of the Medical University of Vienna and pertinent federal regulatory authorities. After a test-retest phase ensuring reliability of local [^11^C]-(+)-PHNO PET imaging procedures (six male HV), 42 HV and 29 antipsychotic-naïve (or minimally exposed) patients with FEP capable of providing informed consent were recruited between 2013 and 2017; HV were required to be of good health based on physical examination, history, ECG, and laboratory results. Exclusion criteria comprised any intake of drugs of abuse except nicotine, caffeine, and alcohol (occasional use only), five or more stimulant exposures lifetime, psychiatric disorders (evaluated with the DSM-IV based M.I.N.I. questionnaire^25^), having a first-degree relative with schizophrenia or bipolar disorder or having any contraindications against receiving a PET, MRI or d-amphetamine; in addition, FEP patients were required to have a minimum Positive and Negative Symptom Scale (PANSS^26,27^) score of 55 with >3 on at least two PANSS psychosis items or >4 on one psychosis item; no or minimal lifetime exposure to antipsychotics; no lifetime exposure to antipsychotic depot preparations; no antipsychotics within two weeks prior to scanning (for details see Supplemental Material). Diagnoses of FEP in SCZ according to DSM-IV were independently made by at least two experienced psychiatrists (N.M.; N.P-R., S.K., M.W.). Three patients were previously exposed to olanzapine, aripiprazole, or quetiapine. Antipsychotics had been discontinued at least two months prior to inclusion without attaining a predefined threshold of two treatment weeks or lifetime exposure up to 50 mg haloperidol-equivalent. Three patients had a history of antidepressant treatment discontinued at least two months prior to inclusion. During the study, eleven patients required symptomatic treatment for psychomotor agitation or insomnia (lorazepam 1–10 mg or zolpidem 10 mg per day). Data sets (complete or partial) of 28 + 6 HV and 21 FEP patients entered final analysis (see Supplemental Material and Supplementary Table 1 for full details).

### Study setup and prospective sensitization

**Fig. 1:**
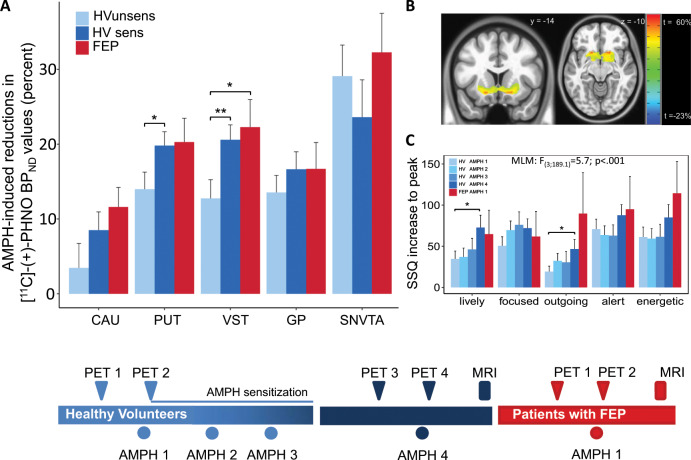
Amphetamine (AMPH)-induced dopamine (DA) release and subjective AMPH effects in healthy volunteers before and after AMPH-sensitization and in drug-free patients with first-episode psychosis (FEP). Upper panel: **a** AMPH-induced DA release in five subdivisions of the basal ganglia (CAU Caudate, PUT Putamen, VST Ventral Striatum, GP Globus Pallidus, SNVTA substantia nigra/ventral-tegmental area) in healthy volunteers before (HV_UNSENS_; sky-blue) and after prospective sensitization to AMPH (HV_SENS_; deep-blue), and in antipsychotic-naïve patients FEP (red). Patients show larger AMPH-induced DA release than HV_UNSENS_. Prospective sensitization of HV_UNSENS_ induced by repeated AMPH administration amplifies DA release, such that the AMPH response in HV_SENS_ no longer differs from FEP. **b** Statistical parametric maps displaying brain areas with the largest sensitization-induced increases in DA-release. Peak effects (up to 60 percent increase) are found in VST (MNI coordinates *x* = −19, *y* = −16, *z* = −10). **c** Subjective AMPH effects in HV undergoing prospective sensitization to AMPH. Repeated administration (four times; light to deep-blue) of AMPH at low constant dose (0.3 mg/kg body weight) successfully induced sensitization as shown by the progressive increase in the AMPH response. Patients with FEP (red; one administration of AMPH) displayed marked AMPH effects already at first contact with the drug. SSQ Subjective States Questionnaire; MNI Montreal Neurologic Institute standard space; bars represent mean ± standard error of the mean; post-hoc two-tailed *t*-tests: *p < 0.05, **p < 0.01). Lower panel: Study flowchart.

First, drug-naïve HV (HV_UNSENS_) underwent an AMPH-free [^11^C]-(+)-PHNO PET scan (baseline_1_, PET_1_). On a separate day less than five 5 days apart, a second scan (PET_2_) was performed 90–120 min after oral administration of 0.4 mg/kg body weight AMPH (Attentin^®^, MEDICE Arzneimittel GmbH, Iserlohn, D), a time point at which subjective effects are peaking and blood levels are still rising (blood levels are highest after 3–4 h^28^). The AMPH dose was chosen according to earlier studies showing that dosages between 0.3 and 0.5 mg/kg body weight induce reliable reductions in [^11^C]-(+)-PHNO BP_ND_ values^29,30^. The dose corresponds to a low to medium dose used for treating attention deficit-hyperactivity disorder in children. Administration of AMPH at this same dose was repeated two more times at intervals of two days. Two to four weeks thereafter, the now sensitized HV (HV_SENS_) underwent another AMPH-free scan (baseline_2_, PET_3_), and within five days, a fourth scan (PET_4_) preceded by AMPH dose four (Fig. 1, lower panel).

Patients with FEP received one baseline scan (PET_1_) and one AMPH-scan (PET_2_; protocol as above). AMPH led to temporary increases in heart rate and blood pressure (Supplementary Fig. 1). Occasionally, participants reported mild headache and insomnia the night after AMPH administration. Neither HV nor patients experienced any serious AMPH-related adverse events.

### [^11^C]-(+)-PHNO PET and MR imaging

[^11^C]-(+]-PHNO was synthesized as described earlier^31^. Quality control was in accordance with European Pharmacopoeia. PET images were acquired on a GE Advance scanner (General Electric Medical Systems, Milwaukee, WI). Emission data were acquired over 90 min after bolus-injection of 309 [81] MBq (mean [SD]) [^11^C]-(+)-PHNO. Raw data were reconstructed by filtered-back projection to yield dynamic images in 15 consecutive one-minute frames followed by 15 five-minute frames. With exception of two patients who chose to terminate their participation early, all subjects underwent T1 and proton density (PD) weighted 3 T magnetic resonance (MR) imaging (see Supplemental Material).

### Behavioral and hormonal measurements

Subjective AMPH effects were recorded using the drug effects questionnaire^32^ and the subjective states questionnaire (SSQ)^33^. The PANSS scale^26^ was administered by certified raters for measuring baseline psychopathology in FEP patients, the brief psychiatric rating scale (BPRS^34^) was used to measure AMPH-induced changes in psychopathology. Blood for serum AMPH levels was collected at the beginning of PET scans. Heart rate, blood pressure (systolic and diastolic were determined repeatedly before and during PET scans (see Supplemental Material).

### Image analysis

#### Analysis in regions of interest

Frame-wise motion correction and co-registration of attenuation-corrected average PET images to T1-weighted MRIs was performed using AFNI software. PET images of two FEP patients who did not undergo MR imaging (see above) were co-registered to normalized [^11^C]-(+)-PHNO template images (one each for no-intervention and AMPH scans) created by averaging spatially normalized PET images (early and late low-contrast frames omitted) of 15 HV. Individually optimized bilateral regions of interest (ROIs) were obtained for the caudate nucleus (CAU), putamen (PUT), ventral striatum (VST), and cortical cerebellum (CER) using the automated image analysis software ROMI^35^ as described elsewhere^30^. Since automated algorithms provided no satisfactory ROI delineation in globus pallidus (GP) and substantia nigra/ventral tegmental area (SNVTA), GP and SN/VTA were delineated manually by a single rater on individual PD MR images (fused with PET images for aiding delineation of SNVTA). ROI delineation was evaluated independently by a second rater visually controlling for anatomical fit and assessing outliers and time drift in ROI sizes. Decay-corrected time–activity curves (TACs) were extracted from the dynamic sequence. The simplified reference tissue model (SRTM2)^36,37^ implemented in PMOD software (Version 3.6; PMOD Technologies Ltd, Zurich, Switzerland) was used to derive binding potential (BP_ND_) values in each ROI. Cerebellar cortex (CER) avoiding midline structures served as reference region since it is virtually devoid of DA D_2/3_ receptors in humans^38–40^. Relative AMPH-induced change in [^11^C]-(+)-PHNO BP_ND_ binding was calculated as [(BP_ND baseline_ – BP_ND AMPH_)/BP_ND baseline_ * 100] (ΔBP_ND_; for the sake of simplicity henceforth designated “DA release”).

#### Parametric analysis

Voxel-wise BP_ND_ maps were calculated using the PMOD 3.6 SRTM basis function implementation^41^. TACs previously derived in CER (low-binding) and VST (high-binding) were used to optimize iterative model fitting procedures. Effects of AMPH and AMPH-sensitization were analyzed using the AFNI programs 3dttest++ and 3dLME.

#### Volumetric analyses

T1-weighted MR images were processed using Freesurfer 6.0 software (http://surfer.nmr.mgh.harvard.edu/). Cortical gray voxels were allocated to a set of regions predefined in the Destrieux atlas^42^ using a Bayesian algorithm. Skull-stripping, gross accuracy of delineation, and surface modeling were quality-controlled by visual inspection of processed images.

### Statistical analysis

**Fig. 2:**
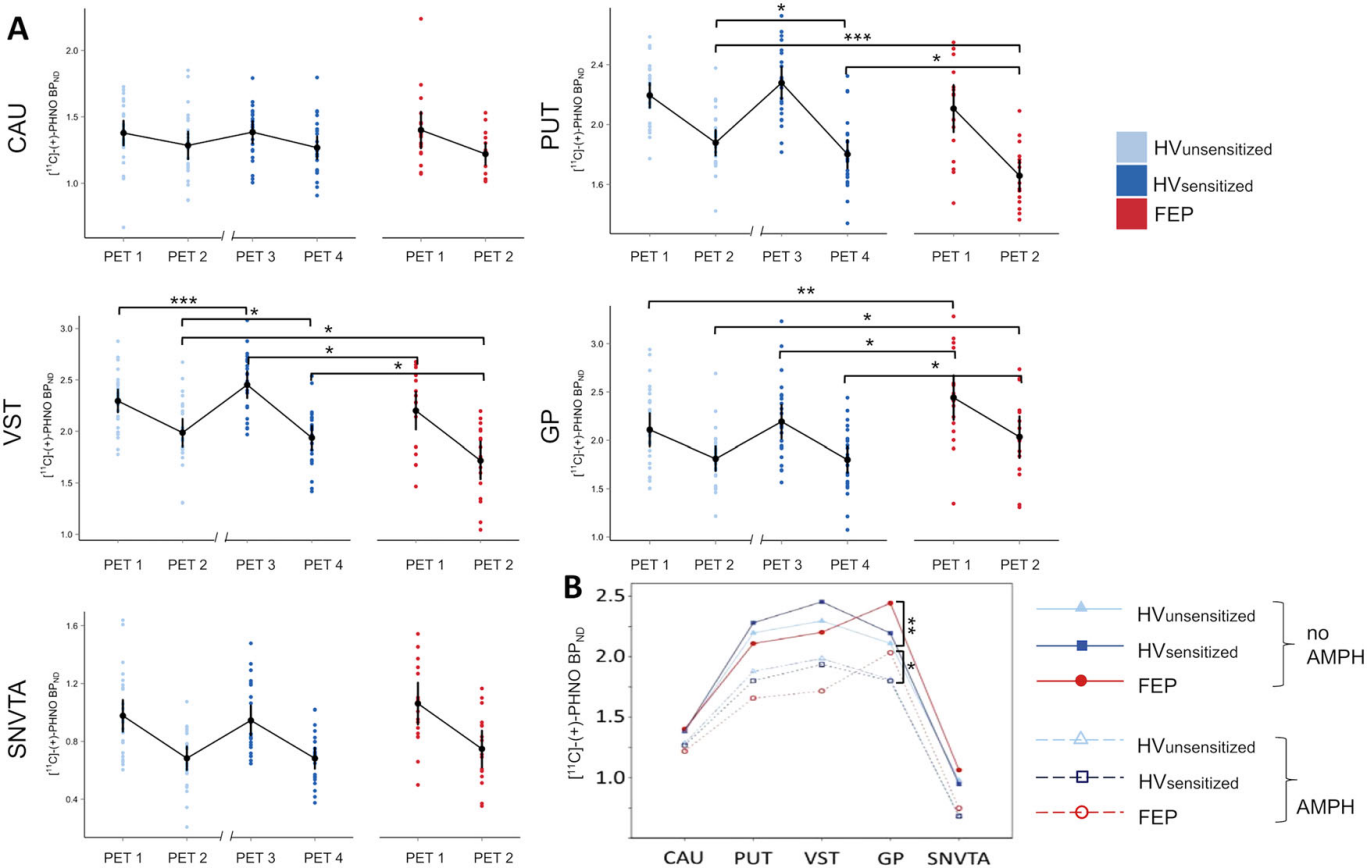
Dopamine (DA) D_2/3_ receptor binding ([^11^C]-(+)-PHNO BP_ND_ values) in scans without (PET1 and PET3) and with prior administration of amphetamine (AMPH; PET2 and PET4). **a** Panels show binding in five subcortical regions of interest (ROIs; CAU, caudate; PUT, putamen; VST, ventral striatum; GP, globus pallidus; SNVTA, substantia nigra/ventral-tegmental area) in healthy volunteers before (HV_UNSENS_; sky-blue) and after AMPH sensitization (HV_SENS_; deep-blue) and in patients with first-episode psychosis (FEP; red). *X*-axis break between PET2 and PET3 in HV indicates a scan-free interval (2–4 weeks) during prospective AMPH sensitization. All direct AMPH effects (PET1 vs. PET2 HV_UNSENS_ and FEP, PET3 vs. PET4 HV_sens_) were significant (paired *t*-tests *p* = 0.03–2.5 × 10^−5^; not marked). Note that AMPH sensitization led to a significant increase in D_2/3_ receptor binding from PET1 to PET3 in VST. **b** Alternative representation of data shown in **a** highlighting systematic differences in D_2/3_ receptor binding across conditions between HV and patients with FEP. D_2/3_ binding in FEP is lower than in HV in *D*_**2**_ receptor-rich neo-striatal regions (CAU, PUT, VST), while it is elevated in *D*_**3**_ receptor-rich regions of the paleo-striatum (GP and SNVTA). Error bars represent 95% confidence intervals. *p < 0.05, **p < 0.01, ***p < 0.001, post-hoc two-tailed *t*-test.

**Fig. 3:**
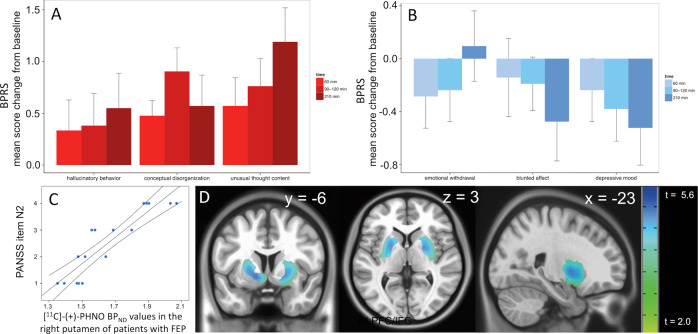
Amphetamine (AMPH)-induced changes in psychopathology and relationship between [^11^C]-(+)-PHNO binding and emotional withdrawal in patients with first-episode psychosis (FEP). **a** Changes in brief psychiatric rating scale (BPRS) positive and **b** negative symptom scores at different time-points after AMPH administration in patients with FEP. Values were calculated as increase or decrease from baseline scores. Bars represent mean ± standard error of the mean. Psychopathological changes did not require medical intervention and returned to baseline levels shortly after PET scanning. **c** Correlation between ‘emotional withdrawal’ and [^11^C]-(+)-PHNO BP_ND_ values in the right putamen of patients with FEP. **d** Voxel-wise analysis showing the correlation between severity of the negative SCZ symptom ‘emotional withdrawal’ (PANSS item N2) and [^11^C]-(+)-PHNO BP_ND_ values in patients with FEP (*p*_corr_ < 0.01; *t*-max: 5.68 at MNI *x* = −21, *y* = 0, *z* = −11).

**Fig. 4:**
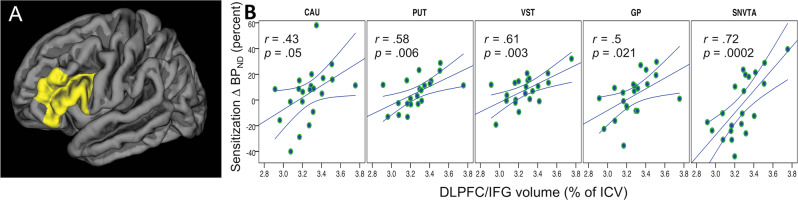
Vertex-wise analysis on the relationship between sensitization in dopamine (DA) release (increase in amphetamine-induced changes in [^11^C]-(+)-PHNO BP_ND_ values) and whole-cortex volumetric measures in healthy volunteers (HV). **a** The analysis identified largest effects in a left-hemispheric cluster comprising the dorsolateral prefrontal cortex (BA46) and Broca’s area (BA 44 and 45; cluster thresholded at t = −4.3; peak signal at MNI coordinates x = 6, y = −13, z = −7). **b** Positive relationship between sensitization of DA release and volume of the inferior frontal gyrus (IFG).

**Fig. 5:**
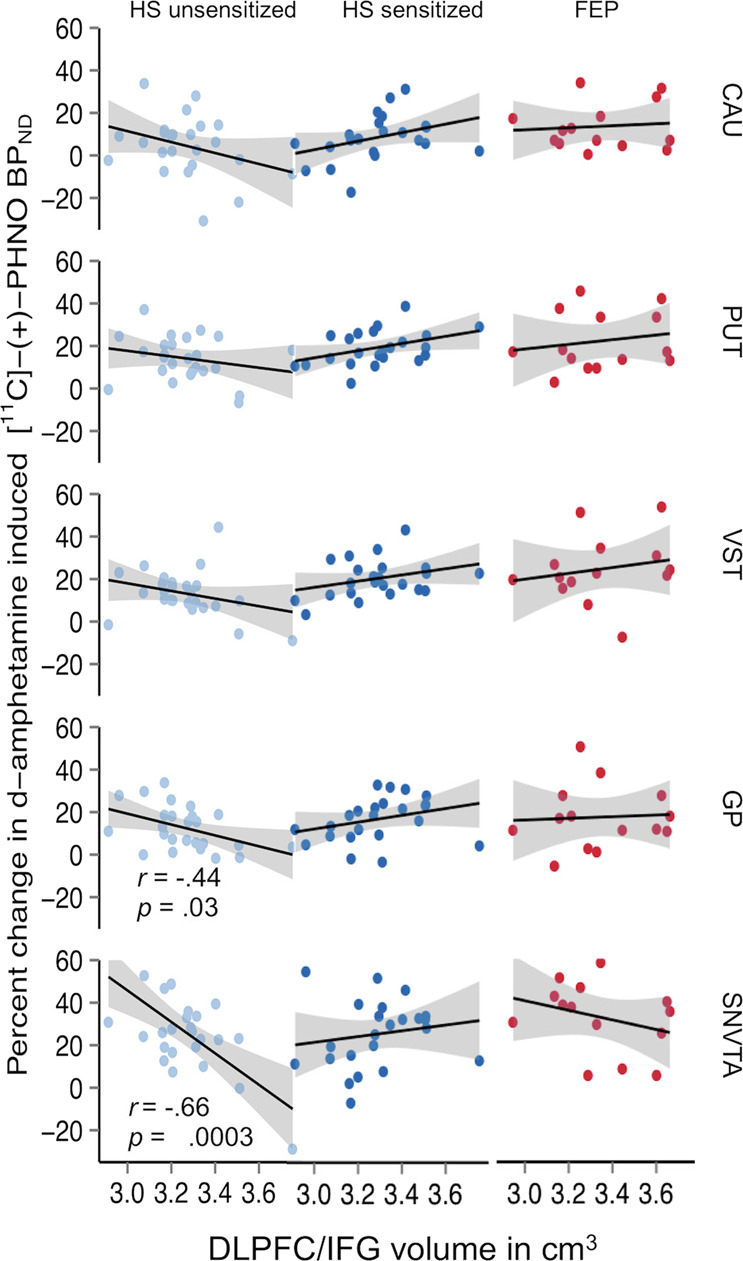
Relationship between volume of the dorsolateral prefrontal cortex (in cm^3^) and inferior frontal gyrus (DLPFC/IFG) as measured with magnetic resonance imaging and AMPH-induced reductions in non-displaceable binding potential (BP_ND_) values of the dopamine *D*_2/3_ receptor positron emission tomography (PET) radioligand [^11^C]-(+)-PHNO in healthy subjects before and after sensitization to d-amphetamine compared to patients with schizophrenia. Significant interactions are found in brain regions (GP, SNVTA) where dopamine *D*_3_ (in contrast to *D*_2_) receptors are the predominant source of signal detected with [^11^C]-(+)-PHNO and PET. VST, ventral striatum; GP, globus pallidus; SNVTA, substantia nigra/ventral tegmental area.

The sample size of this study was planned according to data collected with antagonist radioligands in sensitization and patients with schizophrenia^3–5,15^. Statistical analysis of [^11^C]-(+)-PHNO BP_ND_ values obtained in the ROI-based analysis of [^11^C]-(+)-PHNO binding was carried out using mixed linear models (MLM) as implemented in SPSS 24.0 (SPSS 24.0; IBM Corp., Armonk, NY) and R 12.0.1 (R Foundation for Statistical Computing, Vienna, Austria; https://www.r-project.org) software. Analysis of differences in DA release (ΔBP_ND_) between HV before and after sensitization was carried out using ΔBP_ND_ as dependent variable and group (FEP vs. HV_UNSENS_, and FEP vs. HV_SENS_, respectively) as fixed variable; ROI was entered as random variable. For analyzing receptor binding, BP_ND_ was entered as dependent variable, subject status (HV_UNSENS_, HV_SENS_, FEP) as fixed between-group factor, scan condition (AMPH yes/no) as fixed repeated factor, and ROI as fixed or random repeated factor. MLM analyses testing the effects of covariates (PFC volume parameters) were carried out analogously. All main effects and all relevant interactions were entered into the respective models. The subject-identifier variable was entered as random factor where appropriate. Two-tailed *t*-tests (paired where appropriate) were used for post-hoc tests after assuring normal distribution. Correlations were calculated using Pearson product moment (*r*) or, if appropriate, Spearman rank sum (*rho*) correlation coefficients. Results confirming the main a-priori study hypotheses (Figs. 1, 2) were not corrected for multiple comparisons. More exploratory results (Figs. 3–5) were carried out using ROI-based and parametric methods (voxel-wise maps for PET images, vertex-wise analysis of MR images) for independent confirmation. Results of the ROI-based analysis on the relationship between PANSS items and BP_ND_ values (Fig. 3; 30 PANSS items, 5 ROIs, two hemispheres and on/off AMPH) and the relationship between regional cortical volumes as implemented in Freesurfer 6.0 software^42^ and ΔBP_ND_ (Fig. 4; 33 cortical regions, 5 ROIs, two hemispheres; three groups) were Bonferroni corrected, resulting in adjusted significance levels of *p*_corr1_ = 0.00008 and *p*_corr2_ = 0.00005, respectively.

## Results

### DA release in FEP and sensitization

The concept of “endogenous sensitization” in psychosis^17^ predicts that the difference in AMPH-induced DA release between FEP patients and HV should disappear or substantially diminish after HV are sensitized to AMPH. Analysis of AMPH-induced changes in [^11^C]-(+)-PHNO binding (ΔBP_ND_) showed significant differences between unsensitized HV and FEP (HV_UNSENS_ vs. FEP: *F*_(1;184.04)_ = 11.6, *p* *=* .0008: Fig. 1a). After HV were sensitized, groups were no longer significantly different (HV_SENS_ vs. FEP: *F*_(1;175.6))_ = 0.99, *p* *=* .32; Fig. 1a, Supplementary Table 2). Including alcohol and nicotine consumption and sex as covariates into the model did not relevantly alter results (HV_UNSENS_ vs. FEP: *F*_(1;139.9)_ = 11.6, *p* = 0.0008, see Supplemental Material for details). In good agreement with the ROI-based analysis, parametric ΔBP_ND_ maps showed the most robust effects of sensitization in the VST (Fig. 1b). Indicating behavioral sensitization in HV_SENS_, repeated AMPH-administration induced progressive enhancement of subjective AMPH effects up to levels observed in FEP (Fig. 1c, Supplementary Fig. 2).

Analysis of group differences and AMPH-effects at the level of D_2/3_ receptor binding before sensitization ([^11^C]-(+)-PHNO BP_ND_ values by group, condition, and ROI) showed significant two-way interactions between group and condition (HV_UNSENS_/FEP * no-AMPH/AMPH: *F*_(1;305.7)_ = 9.9, *p* = .009), group and ROI (*F*_(4;140)_ = 10.7, *p* = 1.3 × 10^−7^), and a significant three-way interaction between group, condition, and ROI (*F*_(8;140)_ = 3.0, *p* = .004).

The same model, when applied to FEP and HV after sensitization, did no longer show a significant two-way interaction between group and condition (*F*_(1;275.8)_ = 0.6, *p* = 0.42). However, the group*ROI (*F*_(4;123)_ = 11.2, *p* = 9.4 × 10^−8^), and a group*condition* ROI interactions remained significant, (*F*_(8;123.1)_ = 5.5, *p* = 5 × 10^−5^; Fig. 2a, b). At least in part, this is due to the fact that irrespective of AMPH pretreatment, D_2/3_ receptor binding was consistently lower in FEP than in HVs in neo-striatal ROIs (CAU, PUT, VST) but higher in GP (and trend-wise also in SNVTA; Fig. 2b; Supplementary Table 3). Baseline D_2/3_ receptor binding and sensitization showed a significant positive correlation for all regions in HV (see Supplementary Fig. 3). An analysis of functional subdivisions of the basal ganglia as previously published did not reflect our findings (Supplementary Fig. 4).

Indices of DA release were positively correlated with behavioral AMPH effects (see Supplemental Material). It is known that benzodiazepines might interact with dopamine neurotransmission^43^. Thus we compared DA release between patients receiving benzodiazepines and those who did not require sedation, we found no significant difference (16 available datasets for calculating DA release, 7 not receiving lorazepam, 9 receiving lorazepam; two-sided *t*-test *p* > 0.05 for all ROIs).

### AMPH effects on psychopathology

With a mean [SD] PANSS score of 82.2 [6.9] (subscales for positive, negative, and general SCZ symptoms: 21.4 [6.8], 20.1 [6.1], and 40.8 [9.4]), FEP patients were suffering from acute psychosis of moderate to marked severity at the time of PET scanning. Administration of AMPH prompted a temporary increase in psychotic symptoms. Symptoms returned to baseline after 3–4 h without specific intervention. While positive symptoms of SCZ (in particular hallucinations, delusions, and thought-disorder) increased with AMPH, negative (especially blunted affect and emotional withdrawal) and affective symptoms (depressed mood) improved (Fig. 3a, b; Supplementary Table 4). In contrast to earlier studies^3–6,44–46^ but in line with a recent study using the D_2/3_ receptor agonist radioligand [^11^C]*N*-propyl-apomorphine ([^11^C]NPA^47^), we did not observe significant relationships between positive symptoms and DA release. However, there were significant relationships between [^11^C]-(+)-PHNO BP_ND_ values in the AMPH condition and negative symptoms. Correlations were strongest in PUT and driven mainly by the PANSS item *N*2 (“emotional withdrawal”; right PUT: rho = 0.93, *p* = 7.7 × 10^−8^; Fig. 3c, d; Supplementary Fig. 5). Typical behavioral effects of AMPH in healthy volunteers included increased production of speech, extraversion, alertness, and enhanced energy.

### PFC volume and subcortical DA release

Dysfunctional PFC-DA interactions are prime candidates for “upstream” pathogenic mechanisms underlying subcortical hyperdopaminergia in patients with SCZ. Furthermore, brain circuits involved in cognitive functions encompassing the PFC are often found to be compromised in patients with SCZ^48^. Thus we derived volumetric parameters from MR images and, in a first step, analyzed the relationship between indices of DA release and overall PFC volume. Consistent with inhibition of DA release by the PFC^19,22^, we observed an inverse relationship between PFC volume and DA release in AMPH naïve HV_UNSENS_. After AMPH sensitization, the association was lost or even reversed and more resembled the pattern observed in FEP patients. An anatomically unbiased vertex-wise analysis of the relationship between sensitization of DA release in SNVTA (ΔBP_ND-SENS_ − ΔBP_ND-UNSENS_) and whole-cortex volumetric parameters identified a left-hemispheric cluster spanning the boundaries of middle and inferior frontal gyrus as main driver of the whole-PFC signal (Fig. 4a, b). In a next step, we analyzed correlations between DA release and volumes of individual PFC sub-regions as implemented into Freesurfer 6.0 software according to anatomical and functional priors^42^. In perfect agreement with the vertex-wise analysis, we observed the by far strongest correlations in three anatomically adjacent left-hemispheric regions: Brodmann areas (BA) 46 (dorsolateral prefrontal cortex; DLPFC), and BA 44 and BA 45, or pars opercularis and triangularis of the left inferior frontal gyrus (IFG), together forming “Broca’s area”^49^.

Plotting the correlations between volume of these regions (henceforth referred to as *DLPFC/IFG)* and DA release in the five subcortical ROIs (CAU, PUT, VST, GP, and SNVTA in rostro-caudal anatomical order) revealed a remarkable “herring bone”-like pattern (Fig. 5). This pattern is shaped by negative regression lines—indicating *DLPFC/IFG*-inhibition of DA release in HV_UNSENS_—and positive regression lines observed after the same subjects had undergone AMPH sensitization (HV_UNSENS_ vs. HV_SENS_ vs. FEP: *F*_(2;278.9))_ = 10.14, *p* = .00005). We did not observe any significant relationships between subcortical DA release and cortical volumes in FEP (Fig. 5). To account for possible changes in brain volume induced by AMPH sensitization, we derived volumetric parameters in an independent group of AMPH naïve, sex and age matched HVs. There were no significant differences between our group of HV_SENS_ and the independent HV group (Supplementary Fig. 6). Although of restricted power, these results argue against gross volumetric effects arising from our sensitization procedure.

## Discussion

This study is the first to directly compare AMPH-induced DA release in patients with FEP to DA release in HV before and after prospective AMPH sensitization. Our data confirm the main hypothesis of this work: subcortical DA transmission in patients with FEP is in a state of “endogenous sensitization” towards AMPH. They thus replicate and extend the results of earlier studies that have used *D*_2/3_ receptor antagonist-radioligands for studying DA release in SCZ^3–6^ and the effects of AMPH sensitization in HV^15^. In contrast to these studies, we found no relationship between DA release and change in positive symptoms of SCZ. However, we observed a significant correlation between [^11^C]-(+)-PHNO BP_ND_ values after AMPH in the putamen and negative symptoms, in particular emotional withdrawal. Although the interpretation of this finding is not straightforward, the finding was highly significant and is somewhat supported another [^11^C]-(+)-PHNO study^50^ that found a negative relationship between stress-induced DA release in SNVTA and negative symptoms in individuals with clinical high risk for schizophrenia (and at trend level, also patients with FEP). Together, these results corroborate the notion that reduced DA transmission is a relevant element in the pathogenesis of negative schizophrenic symptoms.

Our results are at odds with those of a recent study using the D_2/3_ receptor agonist radioligand [^11^C]NPA^47^. In contrast to our results and those obtained with D_2/3_ antagonist radioligands^3–6^, the study by Frankle et al. study did not find enhanced AMPH-induced DA release in schizophrenia. Besides differences in clinical variables (as for example a smaller proportion of medication-naïve patients in the Frankle et al. study), the discrepancy could also reflect different properties of the two radioligands. Since the method used in both studies does not allow for reliably distinguishing between the absolute number of receptors and receptor affinity (including changes in apparent affinity induced by competition with endogenous DA^51^), it remains open how these parameters exactly relate to each other in dopaminergic dysfunction of SCZ^52^.

Relationships between volumetric and functional data suggest that AMPH-induced DA release in HV at first exposure to the drug is under inhibitory control of the left-hemispheric *DLPFC/IFG*. At the same time, AMPH sensitization increased DA release to a greater extent in subjects with larger *DLPFC/IFG* volumes. This relationship was particularly pronounced in GP and SNVTA (Fig. 4), where the [^11^C]-(+)-PHNO signal is predominantly reflecting binding to DA D_3_ rather than D_2_ receptor subtypes^53–55^. Since it is unlikely, according to our data obtained in an independent HV sample, that AMPH sensitization had induced major volumetric changes, the “herring bone” pattern most likely reflects a true functional shift in control of subcortical DA release by the *DLPFC/IFG* from inhibition in HV_UNSENS_ towards facilitation in HV_SENS_. This interpretation is supported by rodent data showing that expression of the sensitized state is critically dependent on the integrity of PFC neuronal tissue and its interaction with subcortical D_3_ receptors^56^.

In our study, prospective sensitization of HV led to an increase in DA release to the magnitude observed in FEP. *DLPFC/IFG* volumes, while showing strong correlations to DA release in HV_UNSENS_, did not show any significant relationship to DA release in patients with FEP (Fig. 4). While we cannot rule out that this is due to lack of power, the very same region was found to show abnormal cortical folding in SCZ patients with a mean illness duration of two decades and extensive exposure to antipsychotics^57^. Thus, our data support the long-standing conjecture that the hyperdopaminergic state in SCZ is directly related to a dysfunction in top-down control of subcortical DA transmission by the PFC^22,58^, and they are consistent with the notion that antipsychotics act by rectifying the consequences of upstream pathogenic factors, which persist despite antipsychotic treatment.

Antipsychotic-sensitive hyperlocomotion is a standard measure for sensitization in rodents and a widely accepted animal model of SCZ. Although not formally quantified in this study, the most evident behavioral effect of AMPH in our participants was an increase in quantitative speech production. Thus, while expressing on a motor level in rodents, enhanced DA transmission in human AMPH-sensitive brain circuits seems to affect primarily neuronal functions involved in the processing of language and speech, making speech being the human equivalent to rodent hyperlocomotion. Alterations in cytoarchitecture introduced into the rodent PFC by disrupting the cytoskeleton of dendritic spines increase subcortical DA levels and induce hyper-locomotion via direct projections between the PFC and SNVTA^59^. Similar alterations in the *DLPFC/IFG* of patients with SCZ may cause dysfunctions in hierarchical control within *DLPFC/IFG*—SNVTA–striato-thalamic loops and may be involved in mediating the autonomous (i.e., not subject to will) production of language in auditory verbal hallucinations of patients with SCZ. Simultaneously activated by certain grammatical constructs^60^, altered interactions between the *DLPFC/IFG* and the evolutionarily ancient SNVTA may also be involved in generating the fascinating and inherently grammatical symptom-complex of schizophrenic “ego disturbances”.

As prospective AMPH sensitization in HV successfully mimics the endophenotype of increased DA release observed in “psychotic sensitization”, our data prompt the question why the endophenotype associates with psychotic symptoms in SCZ but is occurring—unless re-exposed to AMPH—without any relevant behavioral effects in HVs. Symptom-provocation studies^61^ and ecological evidence strongly suggest this to be a matter of dose and intensity of exposure: While an escalation of the AMPH dose induces transient psychotic symptoms in psychiatrically healthy subjects^61^, subjects with stimulant-use disorders indeed exhibit disproportionally high rates of psychotic disorders^62^. Interestingly, subjects abusing methamphetamine or cocaine exhibit increased [^11^C]-(+)-PHNO binding in the GP^63,64^. What seems to differentiate AMPH-induced from “endogenous psychotic” sensitization in our data—higher [^11^C]-(+)-PHNO BP_ND_ values in GP—may just be a matter of dose and length of exposure to AMPH.

An inherent limitation of our method is that it does not discriminate the effects of extracellular DA levels from changes in maximal D_2/3_ receptor binding capacity. In addition, although more sensitive towards fluctuations in extracellular DA, the differences between HVs and FEP patients observed with the agonist radioligand [^11^C]-(+)-PHNO are by no means larger than those observed with antagonist radioligands^1–6^. However, this could in part also be a consequence of increased D_2/3_ receptor occupancy due to higher baseline extracellular DA levels in patients with FEP (Fig. 3b). Another limitation might be the fact that some patients required benzodiazepines due to agitation or anxiety. Although there was no significant difference between medicated and unmedicated patients we cannot rule out the possibility that this might be a confounding factor. Furthermore, although sex did not play a role when entered as a covariate, the imbalance between male and female patients in the FEP sample has to be mentioned as a limitation. Lastly, our design has the limitation that we did not expose, mainly for ethical reasons, our FEP patients to a sensitizing regime of repeated AMPH. However, rarely cited earlier work by Strakowski et al.^65^ has shown that FEP patients fail to show a progressive enhancement in the response to repeated AMPH. This suggests that FEP patients, at least at the level of behavior, are already maximally sensitized. Our data support and extend this finding to a neurochemical level. In summary, we feel confident that our work provides the first direct experimental proof for the hypothesis that the pathogenic substrate underlying psychosis in SCZ is indeed a state of “endogenous sensitization” in subcortical DA systems.

## Supplementary information

Supplemental Material
