# Supplementary material for: On the relationship of first-episode psychosis to the amphetamine-sensitized state: a dopamine D2/3 receptor agonist radioligand study
Source: Transl Psychiatry. 2020 Jan 8;10:2. doi: 10.1038/s41398-019-0681-5 (PMC7026156; doi:10.1038/s41398-019-0681-5)
Supplement: Supplementary file 1 — Supplemental Material [file 41398_2019_681_MOESM1_ESM.docx]

**Supplemental Material**

**Study participants**

After screening 70 potential participants, forty-two healthy volunteers (HV; 19 females, 23 males), recruited by advertisements and by word of mouth at the Department of Psychiatry and Psychotherapy (DPP), Medical University of Vienna fulfilled inclusion criteria of the study and gave written informed consent after full explanation of study procedures. Inclusion criteria: age 18-65, good general health, absence of relevant abnormalities in laboratory screening, ECG or vital signs; no regular use of illegal drugs or alcohol abuse based on history and urine drug screen. Exclusion criteria: current substance use disorder (except nicotine), lifetime use of stimulants exceeding 5 or more exposures, pregnancy or breast feeding, known hypersensitivity to sympathomimetics; history of severe head trauma, positive urine drug screen, presence of MRI exclusion criteria; participation in this study would exceed annual radiation dose limits (20mSv). Additional criteria for patients with SCZ: Inclusion: SCZ according to DSM-5; minimum Positive and Negative Syndrome Scale (PANSS) total score of 55 (> 3 on at least two or >4 on one psychosis item; ability to give informed consent. Exclusion: previous oral antipsychotic treatment for more than 2 weeks or previous treatment with antipsychotic depot preparation. Lifetime substance abuse was assessed by questionnaire. Every study participant received a drug urine screening at each study visit. Females of childbearing potential underwent a urine pregnancy test at each study visit. Every participant underwent a structured M.I.N.I. interview (1). Four HV withdrew consent before scanning, three HV stopped study participation due to adverse events unrelated to study procedures; one HV stopped study participation due to relocation. Sixty-three patients with first episode psychosis (FEP) voluntarily seeking treatment at in- and outpatient units of the DPP, community psychiatric services, and extramural specialists (who all had been regularly contacted and informed by the research team on the main in- and exclusion criteria of the study), were pre-screened for eligibility. Thirty-four patients resulted ineligible because of current antipsychotic treatment, co-morbid substance use disorders, inability to tolerate the study-related delay in specific treatment of a few days, or due to inability or unwillingness to provide informed consent. Twenty-nine antipsychotic-naïve or minimally exposed patients (10 female, 19 male) with first-episode psychosis (FEP) signed informed consent. Three patients withdrew consent before scanning. Study participation was stopped before scanning in one patient because of severe clinical exacerbation. For avoiding re-exposure, study participation was stopped in two patients who experienced significant nausea after the first [^11^C]-(+)-PHNO injection. Study participation in two patients was stopped for avoiding excessive delay in specific treatment after repeated radioligand synthesis failure. Thirty-four HV (14 females, 14 males, 6 male HV for test-retest) and twenty-one patients (6 female, 15 male) with FEP completed the study protocol successfully (**Supplementary** **Table 1**). Three patients had previous sporadic exposure to olanzapine, aripiprazole, or quetiapine. However, medication had been stopped at least two months prior to study participation and did not reach the predefined threshold of a maximum of two treatment weeks or lifetime antipsychotic exposure of 50 mg haloperidol-equivalent. Three patients had previous exposure to escitalopram, sertraline, fluoxetine, trazodone, or mirtazapine. For treating psychomotor agitation and sleep disturbances, it was necessary for 11 patients to receive benzodiazepines (up to 10 mg lorazepam or zolpidem 10 mg per day).

| **Supplementary Table 1.** Demographic table. Mean (SD) | | |  |  |
| --- | --- | --- | --- | --- |
|  | Healthy Volunteers | Patients with FEP |  |  |
| Sex | 14 F, 14 M | 6 F, 15 M |  |  |
| Age (years) | 26.3 (2.6) | 25.1 (6.3) |  |  |
| PANSS^a)^ Positive | - | 21.3 (6.7) |  |  |
| PANSS ^a)^ Negative | - | 20.1 (6.1) |  |  |
| PANSS ^a)^ General | - | 40.7 (9.4) |  |  |
| PANSS ^a)^ Total | - | 82.2 (16.9) |  |  |
| Illness duration (weeks) | - | 47.1 (63.4) |  |  |
| Benzodiazepines | - | 11 yes, 10 no |  |  |

^a)^ Positive and Negative Symptom Scale

**Test-retest data**

For determination of test-retest variability, six male HV (age: 26.5 ± 1.5 years) underwent two 90 min [^11^C]-(+)-PHNO positron emission tomography (PET) scans in drug-naïve conditions at least one week apart from each other. Intraclass correlation coefficients (ICC) were calculated as in Shrout et al(2). There were no significant differences in [^11^C]-(+)-PHNO binding potential (BP_ND_) values between the first and the second scan, with *p*-values ranging from 0.3 to 0.96 (two-sided *t*-test). ICC values >0.9 (*p*< 0.05) were indicative of excellent reliability in the putamen (PUT), caudate (CAU), ventral striatum (VST), whereas lesser reliability was found for globus pallidus (GP) and substantia nigra / ventral tegmental area (SNVTA), likely due to the small size of these regions.

**Impact of AMPH on physiological parameters and blood AMPH levels**

In order to identify sensitization effects in physiological responses to AMPH, we compared AMPH-induced increases in blood pressure and heart rate before and after sensitization (see **Supplementary Figure 1**).

Supplementary Figure 1: d-amphetamine (AMPH)-induced increase of heart rate and mean arterial pressure in healthy volunteers (HV) before, and after, sensitization (AMPH 1 and AMPH 2) and in patients with first-episode psychosis (FEP). There was no significant sensitization effect regarding mean blood pressure (MABP) and heart rate after dose 4 compared to dose 1 (paired two-sided *t*-test *t_(26)_* = 1.39, *p* = .18. However, there was a significant difference regarding MABP between patients with schizophrenia and healthy controls after their first *d*-AMPH administration (two-sided *t*-test *t_(44)_* = -2.59, *p* = .013). This effect disappeared after healthy subjects were sensitized to AMPH (two-sided *t*-test *t_(44)_* = -1.06, 79, *p* = .3). Bars denote standard error of the mean.

Amphetamine serum levels were analyzed by gas chromatography-tandem mass spectrometry (GC-MS/MS) using an isotope-labeled internal standard. The analytical procedure was validated according to the EMA guideline on bioanalytical method validation. There was no significant difference between AMPH levels between HV (48.99ng/mL± 21.1 first administration; 58.23ng/mL± 18.17 fourth administration) and patients with FEP (54.15ng/mL± 21.58) at any given time point.

**Subjective and neurochemical AMPH effects in HV and patients with FEP**

To confirm sensitization effects on a behavioral level, DEQ and SSQ self-rating questionnaires were administered to all study participants prior to, and at regular intervals after AMPH ingestion (60, 90-120, and 210 min). In HV, there was a significant effect of condition on AMPH effects (F_3,3_ = 4.51, *p* = .004): Enhanced AMPH effects in patients with FEP were comparable to the sensitized state in HV (see **Supplementary Figure 2**). This is paralleled on a neurochemical level by changes in displacement and BP_ND_, (**Supplementary Table 2 and 3**).

A

**Supplementary Figure 2.** **(A)** In the SSQ items “lively” and “outgoing” we saw a significant sensitization effect in HV (paired two-sided *t*-test *t_(26)_* = -2.43, *p* = .022 and *t_(26)_* = -2.21, *p*= .036 respectively) but no significant difference between FEP patients and HV.  **(B)** There was a significant sensitization effect in healthy volunteers in the DEQ item “want more” (paired two-sided *t*-test *p* = .03, *t* = -2.3). Trend-wise significance was observed in the DEQ item “feel high” when HV on their first dose of amphetamine and patients with FEP were compared (*p* = .067, *t_(34.55)_* = -1.89, two-sided *t*-test).

Legend: AMPH 1-4: First to fourth dose of AMPH administered to healthy volunteers; FEP: patients with first episode psychosis; bars denote standard error of the mean.

B

| **Supplementary Table 2.** Percent reductions in regional [^11^C]-(+)-PHNO BP_ND_ values of healthy volunteers and patients with FEP relative to AMPH-free baseline conditions. Mean (SD) | | | | | | |
| --- | --- | --- | --- | --- | --- | --- |
|  | Healthy Volunteers | | Patients with FEP | *p* values | | |
|  | Unsensitized  (HV***_UNSENS_***_)_  *n= 25* | Sensitized  (HV***_SENS_***_)_  *n= 23* | FEP  *n= 16* | HV***_UNSENS_***  *vs.*  HV***_SENS_*** | HV***_UNSENS_***  *vs.*  FEP | HV***_SENS_***  *vs.*  FEP |
| Caudate Nucleus | 4.67 (13.98) | 8.74 (10.73) | 11.60 (10.47) | 0.28 | 0.1 | 0.42 |
| Putamen | 14.25 (10.31) | 19.55 (8.12) | 20.28 (12.76) | **0.036** | 0.1 | 0.84 |
| Ventral Striatum | 13.29 (10.89) | 20.57 (8.89) | 22.26 (14.8) | **0.005** | **0.03** | 0.58 |
| Globus Pallidus | 12.57 (10.25) | 16.97 (10.28) | 16.71 (14.01) | 0.39 | 0.28 | 0.93 |
| SNVTA | 26.37 (20.01) | 25.53 (15.99) | 30.88 (17.12) | 0.38 | 0.46 | 0.29 |
|  | | | | | | |

| **Supplementary Table 3.** Mean (SD) [^11^C]-(+)-PHNO BP_ND_ values in healthy volunteers (HV) and patients with first episode psychosis (FEP) | | | | | | |
| --- | --- | --- | --- | --- | --- | --- |
|  | Healthy Volunteers | | | | Patients with FEP | |
|  | HV***_UNSENS_***  Baseline  *n= 27* | HV***_UNSENS_***  AMPH  *n= 25* | HV***_SENS_***  Baseline  *n=24* | HV***_SENS_***  AMPH  *n=25* | FEP  Baseline  *n= 17* | FEP  AMPH  *n= 17* |
| Caudate Nucleus | 1.38 (0.23) | 1.28 (0.25) | 1.38 (0.2) | 1.27 (0.20) | 1.4 (0.28) | 1.22 (0.15) |
| Putamen | 2.19 (0.21) | **1.88 (0.21) ^c) d)^** | 2.28 (0.25) | 1.8 (0.23) | 2.13 (0.31) | **1.66 (0.2)^c) d)^** |
| Ventral Striatum | **2.29 (0.28)^a)^** | **1.99 (0.32) ^c) d)^** | **2.45 (0.3)^a)^** | 1.94 (0.28) | 2.22 (0.37) | **1.72 (0.35)^c) d)^** |
| Globus Pallidus | **2.11 (0.43)^b)^** | 1.81 (0.3) | 2.19 (0.41) | 1.8 (0.44) | **2.47 (0.46)^b)^** | 2.03 (0.41) |
| SNVTA | 0.97 (0.26) | 0.68 (0.2) | 0.94 (0.24) | 0.68 (0.25) | 1.07 (0.3) | 0.87 (0.28) |
| ^a)^ Significant difference between baseline_1_ HV***_UNSENS_*** and baseline_2_ HV***_SENS_***; p<0.005  ^b)^ Significant difference between baseline_1_ HV***_UNSENS_*** and baseline FEP; p<0.05  ^c)^ Significant difference between AMPH HV***_UNSENS_*** and AMPH FEP; p<0.05  ^d)^ Significant difference between AMPH HV***_SENS_*** and AMPH FEP; p<0.05 | | | | | | |

**Tests on the possible influence of nicotine and alcohol consumption and sex on main results of the study**:

AMPH-induced dopamine-release in patients with first-episode psychosis vs. unsensitized healthy volunteers:

| **Type III Tests of Fixed Effects^a^** | | | | |
| --- | --- | --- | --- | --- |
| Source | Numerator df | Denominator df | F | Sig. |
| Intercept | 1 | 3,704 | 28,505 | 0,007 |
| HV^UNSENS^ vs^.^ FEP | 1 | 184,043 | 11,564 | 0,001 |
| a. Dependent Variable: AMPH-induced DA release. | | | | |

Results of the same model including sex, alcohol (number of units) and nicotine consumption (number of cigarettes):

| **Type III Tests of Fixed Effects^a^** | | | | |
| --- | --- | --- | --- | --- |
| Source | Numerator df | Denominator df | F | Sig. |
| Intercept | 1 | 3,598 | 28,834 | 0,008 |
| HV^UNSENS^ vs^.^ FEP | 1 | 139,996 | 11,641 | 0,001 |
| a. Dependent Variable: AMPH-induced DA release. | | | | |

**Baseline BP_ND_ change correlates with sensitization in HV**

Changes in [^11^C]-(+)-PHNO BP_ND_ values measured in scans without AMPH pretreatment correlated positively with sensitization of AMPH-induced DA release (**Supplementary Figure 3**).

Supplementary Figure 3. Relationship between changes in [^11^C]-(+)-PHNO BP_ND_ values derived in PET_3_ (baseline sensitized) relative to PET_1_ (baseline sensitized) and sensitization of AMPH-induced DA release (‘baseline change’). Since two interconvertible states of high and low affinity have not been described for DA D_3_ receptor subtypes, increased [^11^C]-(+)-PHNO BP_ND_ values in DA D_3_ receptor-rich regions (GP, SNVTA) are reflecting either increased D_3_ receptor expression induced by sensitization (see also Guillin et al. 2001) or a decrease in steady-state extracellular DA levels.^4^

**Analysis of subdivisions of basal ganglia**

In addition to our conventional analysis we performed a functional subdivision analysis according to Martinez et al 2003 (5). In Supplementary Figure 5 you can see the results of the anaylsis. We performed the analysis in a preliminary fashion while the project was still ongoing, so a limited number of subjects is included. As evident in the plot the data were characterized by high noise, possibly due to overall slightly smaller regions of interest, and did not seem to be reliable, therefore the final analysis was not perfomed with this method. No difference between healthy subjects (presens or sens) or patients showed a significant difference (p<0.05, **Supplementary Figure 4**).

Supplementary Figure 4. Functional subdivision analysis of the regions of interest. By functionally dividing regions of interest according to Martinez 2003 et al, small regions show a lot of noise and therefore do not deliver reliable results.

The functional subdivisions are created by a ROMI algorithm. Briefly, brains are transformed with rigid body transformation into Talairach und Tournoux space. ROMI divides the striatal regions by a line orthogonal to the intra-hemispheric plane going through the anterior commissure to divide the striatal subdivisions into the pre- and postcommissurial parts. Functional divisions are then assembled according to the criteria of Martinez et al.(5) and Mawlawi et al (6).

**Correlation between behavioral parameters and AMPH-induced dopamine release**

There were temporary psychopathological changes following AMPH administration in FEP patients as recorded by the BPRS. Changes usually returned to baseline shortly after the end of scanning (**Supplementary Table 4**). AMPH-induced changes did not require medical intervention. Strongest increases were observed in the items “conceptual disorganization”, “hallucinations” and “unusual thought content”, while we saw strongest decreases in the items “emotional withdrawal”, “depressed mood”, and “blunted affect”. At single item level, changes did not reach level of significance (two-sided *t*-test; **Supplementary** **Table 4**).

| **Supplementary Table 4.** Increases in 18-item Scale Brief Psychiatric Rating Scale (BPRS) scores in patients with FEP after AMPH administration relative to baseline. Max/Min values denote maximal and minimal values under the influence of AMPH. P-values were derived from paired *t*-test comparing baseline scores to maximal (left p-value column) and minimal (right *p*-value column) BPRS scores. Trend-wise significances are bolded. P-values are uncorrected. | | | | | |
| --- | --- | --- | --- | --- | --- |
|  | Baseline | Max value | Min value | *p*-value | |
| 1. Somatic concern  2. Anxiety  3. Emotional Withdrawal*  4. Conceptual Disorganization  5. Guilt Feelings  6. Tension  7. Mannerism and Posturing  8. Grandiosity  9. Depressed Mood*  10. Hostility  11. Suspiciousness  12. Hallucinatory Behavior  13. Motor Retardation*  14. Uncooperativeness  15. Unusual Thought Content  16. Blunted Affect*  17. Excitement  18. Disorientation | 2.76 (1.76)  3.10 (1.76)  2.67 (1.65)  3.43 (1.47)  2.29 (1.74)  3.81 (1.5)  3.71 (1.19)  2.33 (1.77)  2.43 (1.47)  1.19 (0.6)  2.81 (1.91)  2.95 (2.2)  2.1 (1.58)  1.29 (0.78)  4.1 (1.67)  3.43 (1.8)  3.62 (1.5)  1.38 (0.86) | 3.33 (1.85)  3.48 (1.81)  2.86 (1.68)  4.33 (1.59)  2.29 (1.74)  4.38 (1.4)  3.9 (1.37)  2.48 (1.86)  2.43 (1.21)  1.48 (0.93)  3.24 (2.1)  4.1 (1.89)  2.43 (1.72)  1.43 (0.68)  5 (1.58)  3.43 (1.5)  4.48 (1.57)  1.43 (0.98) | 2.52 (1.78)  2.48 (1.75)  2.29 (1.52)  3.9 (1.55)  1.81 (1.44)  3.62 (1.69)  3.52 (1.60)  2.05 (1.77)  1.67 (0.97)  1.24 (0.62)  2.71 (1.95)  3.14 (2.1)  1.9 (1.55)  1.24 (0.62)  4.33 (1.59)  2.81 (1.57)  3.71 (1.52)  1.33 (0.8) | .31  .49  .71  **.06**  1  .21  .63  .80  1  .24  .49  .08  .52  .53  .08  1  .08  .87 | .66  .26  .44  .31  .34  .7  .66  .60  **.06**  .80  .87  .76  .69  .83  .64  .24  .84  .85 |

The relationship between behavioral and neurochemical AMPH effects was analyzed using correlation analyses. For this calculation, the maximum increase in DEQ and SSQ values and indices of DA release (percent change in [^11^C]-(+)-PHNO BP_ND_ values) were first tested for normality. Due to presence of some non-normality, these data were analyzed using Spearman correlation coefficients. Visual inspection of the data showed a positive relationship for most DEQ and SSQ items. However, not all assumptions were confirmed by statistical analysis. In HV there was a positive relationship between feeling high upon AMPH administration and AMPH-induced changes in [^11^C]-(+)-PHNO BP_ND_ values in GP (rho = .43, *p* = .049), while “want more” was positively correlated with AMPH-induced changes in CAU (rho = .44, *p* = .045,). Feeling „outgoing“ showed a positive relationship with AMPH-induced changes in GP (rho = .52, *p* = .02). In patients with FEP, feeling „alert“ was positively correlated with AMPH-induced changes in CAU (rho = .68; *p* = .005), PUT (rho = .69 *p* = .004) and VST [^11^C]-(+)-PHNO BP_ND_ values (rho = .75, *p* = .0014). No correlations with subjective and neurochemical AMPH effects were observed in HV after sensitization.

In a next step, we examined the relationship between changes of the BPRS score upon AMPH ingestion and AMPH-induced DA release in patients with FEP. Data analysis used Spearman’s rank sum correlations due to non-normality as confirmed with Shapiro Wilk testing. DA release in SNVTA correlated trend-wise with item 2 (anxiety; rho = -.48, *p* = .057), item 5 (guilt; rho = -.64, *p* = .007), and with item 6 (tension; rho = -.58; *p* = .019).

We analysed the relationship between dopaminergic parameters and psychotic symptoms by correlating [^11^C]-(+)-PHNO BP_ND_ values post-AMPH with PANSS positive, negative, and general symptoms. Contrary to our expectations and previous publications (3, 4) we did not observe significant correlations with positive symptoms, but observed a positive relationship of [^11^C]-(+)-PHNO BP_ND_ values with negative symptoms (**Supplementary** **Figure 5**).

Supplementary Figure 5. Correlations between Positive and Negative Symptoms Scale (PANSS) negative symptoms and [^11^C]-(+)-PHNO BP_ND_ values after d-amphetamine (AMPH) administration in putamen of patients with first-episode psychosis (FEP). The correlations were significant for the items N2, N3, N4, and for the total negative subscale (rho = .58, *p* = .02).

**Validation of volumetric data**

In most cases, magnetic resonance (MR) imaging used for volumetric analyses were performed after amphetamine (AMPH) sensitization. In order to control for possible volumetric effects of repeated AMPH-administration, we compared volumetric measures in our study sample to an independent sample of AMPH-naïve HV recruited for another MRI study at our department. For this purpose, 28 HV from our sample and 28 sex- and age-matched HV were compared regarding grey matter volume of regions that showed significant correlations with AMPH-induced DA release in our data (dorsolateral prefrontal cortex, pars triangularis, opercularis, and orbitalis of the inferior frontal gyrus; **Supplementary Figure 6**).


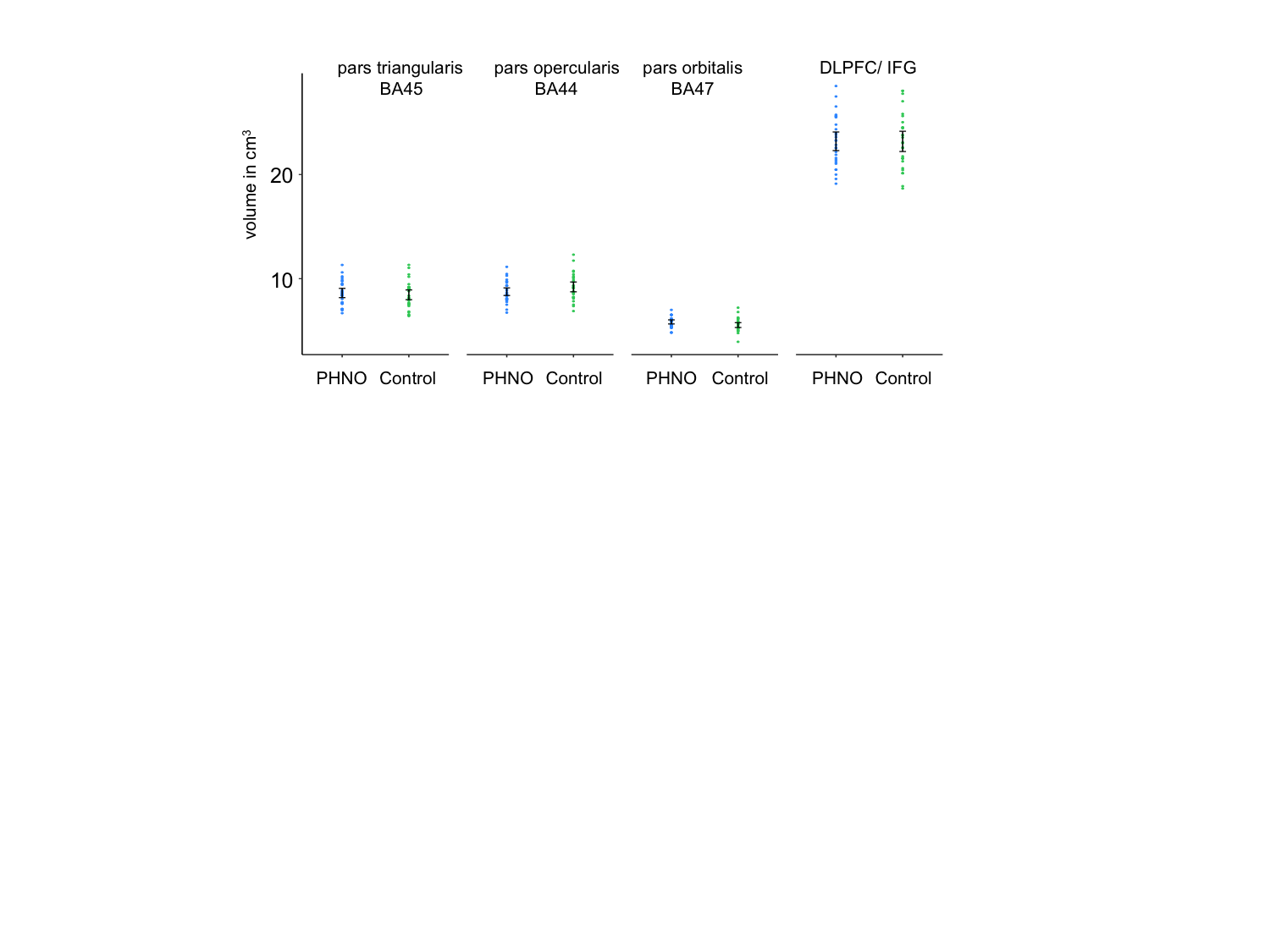


Supplementary Figure 6. Volumes of three frontal brain regions implicated in controlling the response to AMPH in HV who underwent prospective AMPH-sensitization within our [^11^C]-(+)-PHNO PET protocol (PHNO) and 28 age- and sex-matched control HV. MRI volumetric measures showed no significant differences between the two groups, indicating that AMPH sensitization according to our protocol does not induce relevant volumetric alterations in these regions. Thus, in its relationship to parameters of DA release, IFG volume is likely to be cause rather than consequence. Bars denote standard error of the mean.

References

1. Sheehan DV, Lecrubier Y, Sheehan KH, Amorim P, Janavs J, Weiller E, et al. The Mini-International Neuropsychiatric Interview (M.I.N.I.): the development and validation of a structured diagnostic psychiatric interview for DSM-IV and ICD-10. The Journal of clinical psychiatry. 1998;59 Suppl 20:22-33;quiz 4-57.

2. Shrout PE, Fleiss JL. Intraclass correlations: uses in assessing rater reliability. Psychol Bull. 1979;86(2):420-8.

3. Breier A, Su TP, Saunders R, Carson RE, Kolachana BS, de Bartolomeis A, et al. Schizophrenia is associated with elevated amphetamine-induced synaptic dopamine concentrations: evidence from a novel positron emission tomography method. Proceedings of the National Academy of Sciences of the United States of America. 1997;94(6):2569-74.

4. Laruelle M, Abi-Dargham A, van Dyck CH, Gil R, D'Souza CD, Erdos J, et al. Single photon emission computerized tomography imaging of amphetamine-induced dopamine release in drug-free schizophrenic subjects. Proceedings of the National Academy of Sciences of the United States of America. 1996;93(17):9235-40.

5. Martinez D, Slifstein M, Broft A, Mawlawi O, Hwang DR, Huang Y, et al. Imaging human mesolimbic dopamine transmission with positron emission tomography. Part II: amphetamine-induced dopamine release in the functional subdivisions of the striatum. Journal of cerebral blood flow and metabolism : official journal of the International Society of Cerebral Blood Flow and Metabolism. 2003;23(3):285-300.

6. Mawlawi O, Martinez D, Slifstein M, Broft A, Chatterjee R, Hwang DR, et al. Imaging human mesolimbic dopamine transmission with positron emission tomography: I. Accuracy and precision of D(2) receptor parameter measurements in ventral striatum. Journal of cerebral blood flow and metabolism : official journal of the International Society of Cerebral Blood Flow and Metabolism. 2001;21(9):1034-57.
